# Unplanned 30-Day Readmissions After Hospitalization for Irritable Bowel Syndrome

**DOI:** 10.7759/cureus.64519

**Published:** 2024-07-14

**Authors:** Joshua O Ugboke, Fidelis Uwumiro, Efeturi M Okorigba, Ridwan A Lawal, Victory Okpujie, Chukwunonso Ndulue, Love O Temple-Obi, Emmanuel I Bassey, Abdulraheem E Hassan, Sara Ozumba

**Affiliations:** 1 Internal Medicine, College of Medicine, University of Lagos, Lagos, NGA; 2 Internal Medicine, Our Lady of Apostles Hospital, Akwanga, NGA; 3 Internal Medicine, Central Hospital Benin, Benin City, NGA; 4 Internal Medicine, University of Abuja Teaching Hospital, Gwagwalada, NGA; 5 Internal Medicine, College of Health Sciences, University of Uyo, Uyo, NGA; 6 Family Medicine, University of Nigeria, Enugu, NGA

**Keywords:** 30-day readmissions, hospital resource utilization, nationwide readmissions database, ibs (irritable bowel syndrome), irritable bowel disease

## Abstract

Background

Irritable bowel syndrome (IBS) continues to pose significant healthcare challenges due to its broad differential diagnosis and the often extensive yet inconclusive workup. We investigated the rates and characteristics of unplanned 30-day readmissions in adult patients hospitalized with IBS. In addition, we identified factors that predict readmission within 30 days of initial discharge.

Methods

We analyzed the 2020 Nationwide Readmission Database. Using the International Classification of Diseases, Tenth Revision, Clinical Modification code, we identified hospitalizations in adult patients with IBS. We excluded hospitalizations for minors and planned or elective readmissions. To compare baseline characteristics between readmissions and index hospitalizations, χ2 tests were employed. We used multivariate Cox regression analyses to identify independent predictors of readmissions.

Results

A total of 5,729 adult hospitalizations with IBS as the primary diagnosis were discharged alive, and 638 (11.1%) readmissions occurred within 30 days. The most common diagnoses associated with readmission were noninfective gastroenteritis and colitis, sepsis, enterocolitis due to *Clostridium difficile*, and irritable bowel syndrome with or without diarrhea. Patients in readmissions had a mean age of 56.3 years, similar to index hospitalizations (54.5 years, p=0.093). Readmissions had a higher burden of comorbidity (Charlson comorbidity index (CMI) scores ≥3: 26.7%, 170 cases vs. 16.6%, 953 cases; p<0.001) and were mostly Medicare beneficiaries (49.5%, 316% vs. 44.9%, 2,578) compared with index hospitalizations. Readmissions had a longer mean length of stay (LOS) (5.2 vs. 3.6 days, p<0.0001), higher inpatient mortality (0.8%, 5% vs. 0.2%, 11; p=0.032), and higher mean hospital costs ($47,852 vs. $34,592; p<0.0001) compared with index admissions. Secondary diagnoses of ulcerative colitis (adjusted hazard ratio (AHR), 2.82; p<0.0001), interstitial cystitis (AHR, 5.37; p=0.007), peripheral vascular disease (AHR, 1.59; p=0.027), and discharge to short-term hospitals (AHR, 1.03; p<0.0001) were significantly associated with a higher likelihood of readmission within 30 days.

Conclusion

IBS readmissions have poorer outcomes than index hospitalizations. Patients with an existing history of ulcerative colitis, interstitial cystitis, and peripheral vascular disease and those discharged to short-term hospitals following index hospitalization are more likely to be readmitted within 30 days.

## Introduction

Irritable bowel syndrome (IBS), considered a disorder of gut-brain interaction, is a commonly diagnosed gastrointestinal disorder characterized by abdominal pain or discomfort in conjunction with changes in bowel habits, without any underlying causative disease [1]. The diagnosis of IBS has evolved over time, with the Rome IV diagnostic criteria currently being used to identify and confirm cases of IBS. IBS usually involves disrupted bowel patterns (constipation, diarrhea, or a combination) and is accompanied by abdominal bloating or distension. Symptom onset should precede diagnosis by at least six months, and these symptoms must be present within the last three months [2]. In the United States, an estimated 10%-15% of adults experience symptoms associated with IBS. However, the diagnosed prevalence of this condition is notably lower, ranging from 5% to 7% in adults [3]. IBS greatly reduces patients’ quality of life and places a significant resource use burden on the healthcare system to the tune of 1.5 to 10 billion U.S. dollars annually [4]. Other studies have reported data on IBS hospitalizations, but there is a paucity of data on the rates of IBS hospital readmissions in the United States [5-7]. This study analyzed the rates, characteristics, and factors that predict IBS readmissions.

## Materials and methods

Data source

We conducted an analysis using the 2020 Nationwide Readmissions Database (NRD). This database comprises nested and weighted observations that are inherently organized in clusters to provide national estimates. The NRD, maintained by the Agency for Health Care Research and Quality through its Health Care Costs and Utilization Project (HCUP), is accessible online through the HCUP central distributor at http://www.hcup-us.ahrq.gov. The NRD contains comprehensive data on hospital readmissions, including patient demographics (age and sex), hospitalization records (admission and discharge dates, LOS, and discharge status), diagnostic information (primary and secondary diagnoses with ICD codes), procedure codes, hospital characteristics (size, location, and teaching status), readmission indicators (within specified timeframes like 30 days), payer information (Medicare, Medicaid, private insurance, and self-pay), and clinical and utilization data (severity of illness, comorbidities, and healthcare service utilization). This rich dataset enables detailed analysis of patterns and factors associated with hospital readmissions, supporting healthcare outcomes and quality improvement research. The NRD is the largest publicly accessible readmission database in the United States. Unweighted, the 2020 NRD contains data from approximately 17 million discharges. Weighted, it estimates approximately 32 million discharges. The NRD, which encompasses data from the International Classification of Diseases, Tenth Revision, Clinical Modification (ICD-10-CM) for the 2017 dataset, allows for the recording of up to 40 discharge diagnoses per hospitalization. Diagnoses within NRD are categorized into two distinct groups: principal and secondary diagnoses. The principal diagnosis denotes the primary ICD-10 code associated with hospitalization, whereas secondary diagnoses encompass all other ICD-10 codes. Because patient data in the NRD were fully deidentified and publicly available, there was no requirement for institutional review board approval. The NRD serves as a valuable resource for nationwide insights into hospital readmissions. The description of NRD is similar to that in a previous study [8].

Inclusion and exclusion criteria

The NRD captures admissions within a single calendar year without connections to prior or subsequent years. Accordingly, our study encompassed all index hospitalizations of adult patients aged 18 years or older with a primary IBS diagnosis occurring from January to November 2020. Elective or planned readmissions and any readmissions due to trauma were excluded from the analysis. To identify principal and secondary diagnoses, we used the ICD-10-CM diagnostic codes (Table 3 of Appendix).

Outcomes

We computed the rates and reasons for readmissions within 30 days of initial discharge for all index IBS hospitalizations using the weighted NRD sample (both readmissions for IBS and for other causes among the study cohort). Comparisons were made regarding baseline characteristics, inpatient mortality, hospital LOS, and total hospital charges (THCs) between readmissions and initial hospitalizations. We also examined the predictive factors of readmission. For statistical analysis, we used Stata version 17.0MP (StataCorp LLC, College Station, Texas). Baseline characteristics were compared between readmissions and initial hospitalizations using χ2 tests. These characteristics included comorbidities (secondary diagnoses) and categorized variables (insurance type, hospital location, etc.). We accounted for the comorbidity burden using the Charlson comorbidity index (CCI) score. Unadjusted hazard ratios (HRs) for 30-day readmission were determined via univariate Cox regression analysis using patient- and hospital-level variables and comorbidities. All variables with p-values below 0.2 in the univariate analysis were subsequently incorporated into a multivariate Cox regression model. In the multivariate analysis, results are presented as adjusted hazard ratio (AHR) with significance levels set at p<0.05. To address potential confounding variables, we selected factors based on a review of relevant literature.

## Results

A total of 5,742 adult hospitalizations with IBS as the primary diagnosis were analyzed. Among these, 5,729 patients were discharged alive, while 638 (11.1%) readmissions were documented within a 30-day period. During the same period, approximately 2,847,714 hospitalizations occurred due to diseases and disorders of the gastrointestinal system. Therefore, there were two cases of IBS per 1,000 admissions for diseases and disorders of the gastrointestinal tract.

The most common reasons for readmission were noninfective gastroenteritis and colitis (20%, 128 cases), followed by sepsis (16.5%, 105), enterocolitis due to *Clostridium difficile *(12.6%, 80), IBS without diarrhea (11%, 70), IBS with diarrhea (8.3%, 53), unspecified abdominal pain (8.0%, 68), ileus (5.5%, 35), acute pancreatitis without necrosis or infection (5.5% 35), acute kidney failure (2.3%, 15), and gastroparesis (1.5%, 10).

The mean age of patients in readmissions was comparable to that of index hospitalizations (56.3 vs. 54.5 years; p=0.093). Approximately 170 (26.7%) readmissions had a CCI score of three or higher, compared with 953 (16.6%) for index admissions (p<0.001). Significant differences were observed in the prevalence of comorbidities between IBS readmissions and index admissions. These differences included atrial fibrillation (59, 9.3% vs. 316, 5.5%; p=0.003), atrial flutter (6, 0.9% vs. 17, 0.3%; p=0.049), peripheral vascular disease (59, 9.3% vs. 333, 5.8%; p=0.002), hypothyroidism (60, 9.4% vs. 316, 5.5%; p=0.002), congestive heart failure (CHF) (93, 14.6% vs. 482, 8.4%; p<0.001), chronic kidney disease (CKD) (103, 16.1% vs. 614, 10.7%; p<0.002), anemia (87, 13.7% vs. 540, 9.4%; p=0.009), alcohol use disorder (34, 5.3% vs. 161, 2.8%; p=0.003), deep venous thrombosis (10, 1.5% vs. 29, 0.5%; p=0.013), bladder pain syndrome (5, 0.8% vs. 23, 0.4%, p=0.003), and ulcerative colitis (1, 0.2% vs. 17, 0.3%, p<0.001).

Patients with private insurance accounted for 23.4% (149) of readmissions compared with 29.9% (1,717) for index admissions (p=0.011). The mean LOS for index hospitalizations was 3.6 days, whereas that for readmissions was 5.2 days (p<0.001). Approximately 41.3% (263) of readmissions had a moderate loss of function (LOF), compared with 47.1% (2,704) of index hospitalizations (p<0.001). Mortality rates for index hospitalizations and readmissions were low. We found that 11 (0.2%) of the index hospitalizations resulted in inpatient mortality, whereas five (0.8%) of the readmissions resulted in inpatient mortality (p<0.032).

Table 1 presents other baseline characteristics and outcome differences between index hospitalizations and readmissions.

**Table 1:** Comparison of baseline characteristics of IBS hospitalizations and 30-day readmissions Categorical data is presented as counts with percentages, n(%) unless otherwise specified. Numerical data is presented as mean +/- standard deviation. AMA, against medical advice; MI, myocardial infarction; PCI, percutaneous coronary intervention; CABG, coronary artery bypass graft; CCI, Charlson comorbidity index; CHF, congestive heart failure; CKD, chronic kidney disease; DM, diabetes mellitus; PVD, peripheral vascular disease; LOF, loss of function; GERD, gastroesophageal reflux disease; DRG, diagnosis related group; THC, total hospital costs; IBS, irritable bowel syndrome

Variables	Index admissions (N=5,742), n (%) unless otherwise specified	Readmissions (N=638), n (%) unless otherwise specified	p-value
Mean age, years	56.3	54.5	0.093
Inpatient death	11 (0.2)	5 (0.8)	0.032
Female	4,536 (79)	472 (74)	0.017
Mean LOS, days	3.6	5.2	<0.001
Mean THC, US$	34,592	47,852	<0.001
Aggregate hospital costs, US$	199 million	30.4 million	<0.001
Insurance status			
Medicare	2,578 (44.9)	315 (49.5)	0.083
Medicaid	1,005 (17.5)	135 (21.2)	0.064
Private	1,717 (29.9)	149 (23.4)	0.011
Self-pay	269 (4.7)	24 (3.8)	0.419
CCI			<0.001
0	2,595 (45.2)	213 (33.4)	-
1	1,401 (24.4)	150 (23.5)	
2	787 (13.7)	105 (16.5)	
≥3	953 (16.6)	170 (26.7)	
All patients refined DRG severity class			<0.001
Minor LOF	1,470 (25.6)	139 (21.8)	-
Moderate LOF	2,704 (47.1)	263 (41.3)	
Major LOF	1,476 (25.7)	206 (32.3)	
Extreme LOF	97 (1.7)	30 (4.7)	
Admission day is a weekend	1,418 (24.7)	147 (23.0)	0.508
Median household income (quartile)			
First (0-25th)	1,487 (25.9)	175 (27.4)	0.505
Second (26th– 50th)	1,579 (27.5)	160 (25.0)	0.292
Third (51st-75th)	1,424 (24.8)	151 (23.7)	0.613
Fourth (76th-100th)	1,177 (20.5)	142 (22.2)	0.402
Metropolitan hospital	5,409 (94.2)	591 (92.6)	0.264
Teaching hospital	4,105 (71.5)	449 (70.3)	0.627
Hospital bed size			0.723
Small	987 (17.2)	108 (17.0)	-
Medium	1,671(29.1)	142 (22.3)	
Large	3,089 (53.8)	355 (55.7)	
AMA	92 (1.6)	15 (2.4)	0.214
Discharge quarter			0.005
First	1,372 (23.9)	108 (16.9)	-
Second	1,648 (28.7)	183 (28.7)	
Third	1,591 (27.7)	186 (29.2)	
Fourth	1,131 (19.7)	161 (25.2)	
Hospital ownership			0.541
Government	447 (7.8)	50 (7.8)	-
Private, not-for-profit	4,249 (74.0)	458 (71.8)	
Private, investor-owned	1,045 (18.2)	130 (20.4)	
Resident of same state as hospital	5,507 (95.9)	615 (96.4)	0.634
GERD	1,958 (34.1)	248 (38.8)	0.058
Celiac disease	69 (1.2)	0 (0.0)	0.074
Functional dyspepsia	40 (0.7)	0 (0.0)	0.115
Fibromyalgia	40 (0.7)	0 (0.0)	0.114
Ulcerative colitis	17 (0.3)	1 (0.2)	<0.001
bladder pain syndrome	23 (0.4)	5 (0.8)	0.003
Chronic pelvic pain	40 (0.7)	0 (0.0)	0.115
Dyslipidemia	1,361 (23.7)	174 (27.2)	0.125
Old MI	167 (2.9)	29 (4.5)	0.071
Old PCI	200 (3.5)	32 (5.0)	0.183
Old CABG	115 (2.0)	19 (3.0)	0.201
Old pacemaker	103 (1.8)	14 (2.2)	0.472
Atrial fibrillation	316 (5.5)	59 (9.3)	0.003
Atrial flutter	17 (0.3)	6 (0.9)	0.049
COPD	574 (10.0)	80 (12.6)	0.118
Carotid artery disease	11 (0.2)	3 (0.5)	0.194
Old stroke	287 (5.0)	41 (6.4)	0.208
Hypertension	2,113 (36.8)	232 (36.4)	0.860
PVD	333 (5.8)	59 (9.3)	0.002
Hypothyroidism	316 (5.5)	59 (9.3)	0.002
DM type 1 and 2	672 (11.7)	85 (13.3)	0.352
Obesity	517 (9.0)	52 (8.2)	0.626
CHF	482 (8.4)	91 (14.6)	<0.001
CKD	614 (10.7)	103 (16.1)	0.002
Liver disease	500 (8.7)	54 (8.5)	0.877
Oxygen dependence	86 (1.5)	13 (2.1)	0.381
Electrolyte disturbance	46 (0.8)	6 (0.9)	0.907
Smoking	80 (1.4)	3 (0.4)	0.065
Anemia	540 (9.4)	87 (13.7)	0.009
Alcohol use disorder	161 (2.8)	34 (5.3)	0.003
Sepsis	23 (0.4)	3 (0.5)	0.799
Depression	1,516 (26.4)	193 (30.3)	0.135
Deep venous thrombosis	29 (0.5)	10 (1.5)	0.013

Thirty-day readmissions resulted in a total of 3,282 hospital days. The mean total healthcare costs (THCs) were US$34,592 for index hospitalizations vs. US$47,852 for readmissions (Table 1). Readmissions had US$13,269 (p<0.001) higher mean total hospital cost (THC) compared with index hospitalizations. The aggregate THC for 30-day readmissions was US$30,400,000 (Table 1).

In the multivariable analysis, secondary diagnosis of ulcerative colitis (AHR, 2.82; p<0.0001), interstitial cystitis (AHR, 5.37; p=0.007), peripheral vascular disease (AHR, 1.59; p=0.027), as well as discharge to short term hospitals (AHR, 1.03; p<0.0001) were significantly associated with increased likelihood of readmission within 30 days. Routine home discharge (AHR, 0.32; p<0.0001), discharge to home healthcare (HHC) (AHR, 0.38, p=0.006) or other facilities (AHR, 0.33; p=0.004), and short LOS of one to two days in the index hospitalization (AHR, 0.70; p=0.007) was significantly associated with a lower likelihood of readmission (Table 2). Additionally, carotid artery disease (AHR, 0.3; p<0.0001), sepsis (AHR, 0.30; p<0.0001), and pulmonary embolism (AHR, 0.60; p<0.0001) were found to be associated with lower odds of readmissions (Table 2).

**Table 2:** Univariate and multivariate Cox regression models of predictors of 30-day readmissions for IBS hospitalizations Results of multivariate Cox regression are presented as HR or AHR with accompanying p-values. **Only variables with p<0.2 on univariable analysis were added to the multivariable Cox regression analysis HR, hazard ratio; LOS, length of stay; HHC, home healthcare; AMA, against medical advice; MI, myocardial infarction; PCI, percutaneous coronary intervention; CABG, coronary artery bypass graft; LOF, loss of function; GERD, gastroesophageal reflux disease; HMO, health maintenance organization; CCI, Charlson comorbidity index; DRG, diagnosis related group; COPD, chronic obstructive pulmonary disease; PVD, peripheral vascular disease; CHF, congestive heart failure; CKD, chronic kidney disease; IBS, irritable bowel syndrome; THC, total hospital charge; AHR, adjusted hazard ratio

Variables	Univariable Cox regression		Multivariable Cox regression**	
	Unadjusted HR	p-value	AHR	p-value
Female	0.74	0.015	0.78	0.060
Age				
Age ≥18 and <40	0.79	0.072	0.97	0.868
Age ≥40 and <60 y	1.07	0.604	-	
Age ≥60 y	1.13	0.293	-	
No. of procedures				
0-1	1.11	0.393	-	
≥2	0.90	0.393	-	
Discharge disposition				
Routine discharge	0.60	<0.0001	0.32	<0.0001
Discharge to a short-term hospital	3.09	0.071	1.03	<0.0001
Discharge to other facilities	1.39	0.110	0.33	0.004
Discharge to HHC	1.52	0.011	0.38	0.006
Discharged AMA	2.17	0.013	1	(omitted)
Discharge quarter				
First quarter	Reference	Reference	-	
Second quarter	1.09	0.541	-	
Third quarter	1.04	0.774	-	
Fourth quarter	1.20	0.290	-	
Hospital control				
Government	Reference	Reference	Reference	Reference
Private, not-for-profit	1.19	0.395		
Private, investor-owned	1.48	0.077	1.54	0.070
Insurance status				
Medicare	Reference	Reference	Reference	Reference
Medicaid	1.15	0.327	1.39	0.050
Private including HMO	0.71	0.011	0.94	0.705
Self-pay	0.70	0.026	0.90	0.713
CCI				
0	Reference	Reference	Reference	Reference
1	1.02	0.894		
2	1.38	0.046	0.73	0.157
≥3	1.68	<0.0001	0.69	0.143
All-patient-refined DRG severity class				
Minor LOF	Reference	Reference	Reference	Reference
Moderate LOF	1.34	0.056	1.14	0.400
Major LOF	1.87	<0.001	1.29	0.161
Extreme LOF	2.12	0.066	1.16	0.731
Median household income (quartile)				
First (0-25th)	Reference	Reference	-	
Second (26th-50th)	0.86	0.332	-	
Third (51st-75th)	0.90	0.502	-	
Fourth (76th-100th)	1.02	0.876	-	
Metropolitan hospital	0.83	0.444	-	
Teaching hospital	0.97	0.811	-	
Admitted on a weekend	1.03	0.851	-	
Hospital bed size				
Small	Reference	Reference	-	
Medium	1.05	0.775	-	
Large	0.99	0.972	-	
Discharge quarter				
First	Reference	Reference	-	
Second	1.01	0.969	-	
Third	1.04	0.758	-	
Fourth	0.94	0.675	-	
Hospital ownership				
Government	Reference	Reference	-	
Private, not-for-profit	0.94	0.688	-	
Private, investor-owned	1.20	0.353	-	
LOS				
1-2 days	0.63	<0.0001	0.70	0.007
3-5 days	1.15	0.203	-	
≥6 days	1.65	<0.0001	1.19	0.273
THC	1.04	<0.0001	1.00	0.016
Resident of the same state as hospital	1.03	0.921	-	
GERD	1.14	0.262	-	
Celiac disease	0.22	0.128	0.24	0.163
Functional dyspepsia	0.58	0.576	-	
Fibromyalgia	0.58	0.576	-	
Ulcerative colitis	0.03	<0.0001	2.82	<0.0001
Interstitial cystitis	3.44	0.056	5.37	0.007
Chronic pelvic pain	1.32	0.576	-	
Dyslipidemia	1.09	0.477	-	
Old MI	1.75	0.028	1.33	0.332
Old PCI	1.15	0.621		
Old CABG	1.66	0.095	1.22	0.571
Old pacemaker	1.76	0.083	1.08	0.834
Atrial fibrillation	1.82	0.002	1.30	0.196
Atrial flutter	1.93	0.353	-	
COPD	1.61	0.002	1.36	0.077
Carotid artery disease	0.09	<0.0001	0.30	<0.0001
Old stroke	1.42	0.107	1.12	0.616
Hypertension	1.21	0.087	1.26	0.088
PVD	1.80	0.002	1.59	0.027
Hypothyroidism	1.80	0.002	1	(omitted)
DM type 1 and 2	1.34	0.053	1.41	0.055
Obesity	0.85	0.411	-	
CHF	1.80	<0.0001	1.32	0.201
CKD	1.48	0.008	1.47	0.090
Liver disease	1.31	0.114	1.42	0.056
Oxygendependence	0.61	0.422	-	
Electrolyte disturbance	0.05	<0.0001	0.50	<0.0001
Smoking	1.11	0.845	-	
Anemia	1.46	0.023	1.35	0.080
Alcohol use disorder	1.79	0.013	1.30	0.300
Sepsis	0.09	<0.0001	0.30	<0.0001
Depression	0.99	0.926	-	
Deep venous thrombosis	1.34	0.688	-	
Pulmonary embolus	0.09	<0.0001	0.60	<0.0001

## Discussion

In this study of 30-day readmissions after hospitalization for IBS, a total of 5,742 index hospitalizations occurred in 2020. This corresponded to approximately two IBS admissions per 10,000 hospitalizations in 2020 and two per 1,000 hospitalizations related to gastrointestinal system diseases and disorders. The readmission rate for individuals with IBS was determined to be 11.1%.

The primary cause for readmission was identified as noninfective gastroenteritis and colitis, followed by sepsis, enterocolitis due to *Clostridium difficile*, and various IBS-related conditions. Infectious gastroenteritis is a known causative factor for IBS (known as postinfectious IBS or PI-IBS) representing up to 36% of infectious gastroenteritis. Patients who have had an episode of infectious gastroenteritis are reported to have a significantly increased risk of developing IBS and other functional gastrointestinal disorders compared to uninfected subjects [9]. The association between noninfectious gastroenteritis and IBS is an interesting finding that has not been thoroughly explored in the literature but hints at the relationship between IBS and inflammation of the gastrointestinal tract. Patients with *Clostridium difficile* infection also have a high risk of developing post-infectious IBS, particularly those with a longer duration of infection, anxiety, and higher BMI [10,11].

The index study highlighted various demographic and clinical characteristics associated with readmissions. Despite the mean age of patients in readmissions being comparable to index hospitalizations, there were notable differences in comorbidity burden, with higher CCI scores among readmissions. This heightened comorbidity burden might contribute to the propensity for multiple readmissions and increased resource utilization as reported in other IBD [12]. There is ample evidence in the literature supporting the increased severity of IBS symptoms among patients with multiple co-occurring comorbidities including psychological disorders [13-15]. Significant variations were observed in the prevalence of specific comorbidities between readmissions and index hospitalizations in this study. These differences, including atrial fibrillation, CHF, CKD, and hypothyroidism, could hint at potential causative links that could influence the likelihood of readmission. These findings necessitate further investigation into the potential mechanisms and specific care approaches needed to manage both IBS symptoms and these comorbidities to reduce readmission rates effectively. Recently, cannabis use has been associated with reduced 30-day readmissions from all causes. Cannabis use was, however, not correlated with IBS-specific readmission [16]. Cannabis use may also decrease inpatient healthcare utilization in IBS patients [17].

The study also unraveled differences in hospitalization outcomes, including longer hospital stays and higher THCs for readmissions compared to index hospitalizations. In a related study investigating the socioeconomic impact of IBS in the outpatient setting, the factors associated with higher direct costs included older age, unemployment, IBS subtypes other than constipation, lower disease-specific quality of life, and more severe depressive symptoms. Indirect costs were comprised of absenteeism, presenteeism, and productivity loss due to unpaid labor. These costs were significantly linked to being male and experiencing more severe depressive symptoms [18]. Inpatient care, excluding the cost of prescription medication, has been reported to account for up to 13.6% of all annual IBS healthcare expenditures [19]. With a higher proportion of males and depressed patients in readmissions than index hospitalizations, the findings of the index study support previous reports and further suggest that IBS readmissions contribute substantial proportions to annual healthcare expenditure for IBS care.

The multivariable analyses provided further insights into other factors influencing readmissions related to IBS. Secondary diagnoses such as ulcerative colitis, interstitial cystitis, and peripheral vascular disease were significantly associated with a higher likelihood of readmission. Conversely, certain discharge dispositions, such as routine home discharge and discharge to HHC, were associated with lower odds of readmission. These findings hint at the importance of care for multimorbidity in IBS patients and proper post-hospitalization care arrangements in minimizing readmissions. Other comorbidities such as carotid artery disease, sepsis, and pulmonary embolism were identified as factors associated with lower odds of readmissions. This raises important questions regarding potential protective mechanisms these conditions might offer against IBS-related readmissions, warranting further exploration.

Misdiagnosis of IBS remains a significant challenge in medical practice due to its symptom overlap with other gastrointestinal disorders, such as IBD, celiac disease, and gastrointestinal infections [20-22]. The lack of definitive diagnostic tests for IBS often leads to reliance on symptom-based criteria, which can be subjective and vary among patients. Additionally, the variability in symptom presentation and severity further complicates accurate diagnosis. Misdiagnosis can result in inappropriate treatments, delayed management of the actual underlying condition, increased healthcare costs, and diminished quality of life for patients. Enhancing diagnostic accuracy through improved clinical guidelines, greater awareness, and the use of advanced diagnostic tools is essential to address this prevalent issue.

Strengths and limitations

This study possesses several notable strengths. It draws its population from the largest hospital-based multi-payer registry, lending robustness to the findings. Additionally, the study conducts a comprehensive and in-depth evaluation of the impact of IBS and its subsequent readmissions on the US healthcare system, elucidating the resource burden of IBS-related readmissions. However, certain limitations warrant acknowledgment. Notably absent from the NRD database are factors such as race, medication adherence, physician assessments, and the severity of IBS, which could influence readmission rates. The retrospective nature of the NRD introduces challenges in establishing causal relationships and adjusting residual confounding. The database exclusively presents hospitalization data without specifying individual patients, potentially inflating instances of multiple readmissions. The utilization of ICD-10 codes in the NRD also exposes it to potential coding errors. Despite the established link between infectious gastroenteritis and the subsequent risk of developing functional gastrointestinal disorders, there is a lack of definitive studies demonstrating an increased risk of enteritis in patients with IBS. Considering this gap in research, along with the high rate of misdiagnosis among IBS patients, the role of enteritis in IBS readmissions in the index study should be interpreted with caution.

Despite these constraints, the analytical methods employed, coupled with the substantial sample size, contribute significantly to shedding light on a relatively underexplored subject, while also fostering discourse and paving the way for future controlled, multicenter prospective investigations in this area.

## Conclusions

IBS is characterized by recurrent symptom exacerbations and a tendency for repeated hospital visits. According to the 2020 NRD data, 11.1% of hospitalizations with IBS as the primary diagnosis resulted in readmissions within 30 days. These IBS-related readmissions were linked to higher mortality rates, prolonged hospital stays, and increased total hospital expenses. Patients initially hospitalized for IBS, along with comorbid conditions like ulcerative colitis, interstitial cystitis, and peripheral vascular disease, as well as those discharged to short-term hospitals, have a higher likelihood of readmission. The systematic identification and proactive management of high-risk IBS patient groups have the potential to improve outcomes and curtail the associated healthcare costs linked to IBS care.

## Appendices

**Table 3:** ICD-10-CM diagnostic codes used in the study MI, myocardial infarction; PCI, percutaneous coronary intervention; CABG, coronary artery bypass graft; COPD, chronic obstructive pulmonary disease; DM, diabetes mellitus; CHF, congestive heart failure; CKD, chronic kidney disease; ICD-10-CM, International Classification of Diseases, tenth Revisions, Clinical Modification; DM, diabetes mellitus; GERD, gastroesophageal reflux disease

ICD-10 codes	Code description
K58	IBS
Comorbidities	
K21	GERD
K90	Celiac disease
K30	Functional dyspepsia
M79.7	Fibromyalgia
K51	Ulcerative colitis
N30	Interstitial cystitis
R10.2	Chronic pelvic pain
E78	Dyslipidemia
I252	Old MI
Z9861	Old PCI
Z951	Old CABG
Z950	Old pacemaker
I480, I4811, I4819, I4820, I4821, I4891	Atrial fibrillation
I483, I484, I4892	Atrial flutter
J41, J42, J43, J44	Chronic obstructive pulmonary disease
I652	Carotid artery disease
I63, Z8673	Old stroke
I10	Hypertension
I739	Peripheral vascular disease
E03	Hypothyroidism
E10, E11	Diabetes mellitus type 1 and 2
E660, E6601, E6609, E661, E662, E668, E669	Obesity
I50	CHF
N18	CKD
K70, K71, K72, K73, K74, K75, K76, K77	Liver disease
E870, E871, E872, E873, E874, E875, E876	Electrolyte derangement
Z992	Maintenance dialysis
Z9981	Oxygen dependence
Z87891, F17200	Smoking
D50, D51, D52, D53, D55, D56, D57, D58, D59, D60, D61, D62, D63, D64	Anemia
F32, F33	Depression
A40, A41	Sepsis
F10	Alcohol-related disorders
I82	Deep venous thrombus
I26	Pulmonary embolus
